# Perceptions of orthopaedic medicine students and their supervisors about practice-based learning: an exploratory qualitative study

**DOI:** 10.1186/s12909-022-03771-3

**Published:** 2022-10-05

**Authors:** Muhamed Nsubuga, Robert O Opoka, Moses Galukande, Ian G. Munabi, Aloysius G. Mubuuke, Sarah Kiguli

**Affiliations:** 1 Mulago National Referral Hospital, Kampala, Uganda; 2 School of Medicine, College of Health Sciences, Makerere University, Makerere, Uganda; 3 School of Biomedical Sciences, College of Health Sciences, Makerere University, Makerere, Uganda

**Keywords:** *Practice-based learning*, Orthopaedic *medical students*, *Supervisor*

## Abstract

**Background:**

Practice-based learning is crucial in forming appropriate strategies for improving learning among the medical students that support the country’s understaffed health sector. Unsatisfactory learning consequently results in poor performance of students and poor quality of health care workforce in the long run. Exploring the perceptions about the current practice-based learning system and how to improve is thus vital. This study set out to explore the perceptions of Orthopaedic medicine students and their supervisors about practice-based learning at a tertiary training hospital.

**Methods:**

This was an exploratory phenomenological qualitative study that involved in-depth interviews among 10 Orthopedic students during their rotation in the emergency ward of Mulago hospital and 6 of their supervisors. Interviews were audio-recorded, transcribed, and then imported into Atlas ti 8.3 for analysis. The data were coded and grouped into themes relating to perceptions of practice-based learning, general inductive analysis was used. The general inductive approach involved condensing the raw textual data into a brief and summary format. The summarized format was then analyzed to establish clear links between the perceptions of practice-based learning and the summary findings derived from the raw data.

**Results:**

The mean age of the students was 23 ± 1.5 years. Four out of the six supervisors were Orthopaedic officers while the remaining two were principal Orthopaedic officers, four out of the six had a university degree while the other two were diploma holders. The main themes arising were hands-on skills, an unconducive learning environment, the best form of learning, and having an undefined training structure. Particularly, the perceptions included the presence of too many students on the wards during the rotation, frequent stock-outs of supplies for learning, and supervisors being overwhelmed caring for a large number of patients.

**Conclusion:**

Barriers to satisfactory practice-based learning were overcrowding on the wards and insufficient training materials. To improve practice-based learning, adequate learning materials are required and the number of students enrolled needs to be appropriate for the student – supervisor ratio.

## Background

Practice-based learning is a form of learning that involves being facilitated to transfer theory into the workplace through situated and experiential learning possibly in collaborative groups [1].

The rationale of practice-based learning is embedded in enabling students to have a deeper understanding and the cognitive skills related to patient management rendering them to perform much better through authentic experience and becoming part of a community of practice [2]. Practice-based learning permits trainee medical students to witness progress towards its set objectives by providing platforms for them not only to be self-directed but to also become more experienced [3]. As compared to conventional methods of teaching medical students, particularly lecture-based curricula, practice-based learning does not only encourage appropriate qualities in students but also imparts life-long respect for learning [4].

Besides understanding the different factors, practice-based learning is crucial in forming appropriate strategies for improving learning among the medical students that support the country’s understaffed health sector [5]. In Mulago National Referral Hospital, the reports from management meetings show unsatisfactory learning levels demonstrated by limited skills amongst students at wards during their practice [6].

In Uganda, huge numbers of medical students graduate each year but a number of them have been reported to be under-skilled [7]. Researchers have previously shown that medical students however contribute significantly to the delivery of health care services in clinical settings in teaching hospitals [8]. The participation of the students in health service delivery even makes them gain more practical knowledge and skills [9].

The case for practice-based learning is pertinent in contributing significantly to improved learning amongst medical students and in this arena where the health sector is faced with work overload due to understaffing, it should not be compromised in any way [10]. Mulago National Referral Hospital, the teaching hospital for Orthopaedic students is reported to have a high prevalence of fractures 38.6% [11]. This overwhelms the Orthopaedic department which is low on staffing [12]. The introduction of students to the department can offset this discrepancy through practice-based learning. However, grey literature indicates that the ratio of Orthopaedic students to their supervisors in the Mulago emergency ward is approximately 20:1. This may affect the transfer of skills to the students due to the reduced contact hours with the supervisors, and the quality of their outputs ultimately affecting practice-based learning objectives.

Diligent training of Orthopaedic students is important as far as the provision of health care services and contributing to the health status of the public is concerned. This is mainly because well-trained Orthopaedic students will eventually give a hand to the few healthcare staff in case handling which would otherwise be impossible for healthcare staff alone. It is therefore important to assess empirical knowledge on the factors affecting practice-based learning among Orthopaedic students to identify gaps to improve the services provided by them successfully.

If not addressed, unsatisfactory learning will consequently result in poor performance of the students and poor quality of health care workforce in the long run. While understanding the factors affecting this approach to teaching Orthopaedic students was relevant, exploring the current situation of this system and how to improve it for better outcomes was very important. This study therefore aimed at establishing the practices and factors affecting practice-based learning among Orthopaedic medicine students attending the emergency ward at Mulago National Referral Hospital.

## Methods

### Design and setting of the study

This was an exploratory qualitative study conducted at the emergency department of Mulago National Referral Hospital in Uganda. The emergency department brings tertiary-level care to the communities it serves. Through its affiliation with Makerere University, it also serves as a teaching hospital by providing a full complement of residency programs, undergraduate, Allied Health Professions, and Nurses. To serve the growing needs of the community, this department provides a full range of inpatient and outpatient surgical services. This ward has 135 Orthopaedic students according to the attendance records. The department has a turn-up of an average of 1500 and 1010 patients as outpatients and admissions respectively every month [13]. Therefore, the total number of patients seen in this department annually is 18,000 and 12,120 as outpatient and admissions respectively [13].

### Study population

Orthopaedic students attending the emergency ward and their supervisors at Mulago National Referral Hospital during the study period and who met the eligibility criteria.

### Eligibility criteria

The study included Orthopaedic students stationed at Mulago hospital emergency department and their supervisors both of whom had given informed consent. Students in the year I and those who had missed 50% of time allocated to emergency department attachment were excluded.

### Sample size and sampling

In total, 16 interviews; 6 with supervisors and 10 with students were conducted. Homogeneous purposive sampling was used to select students and supervisors to participate in interviews [14]. We assumed that since the students were pursuing the same school degree, they were similar in the majority of the work-related aspects, and so were the instructors [14]. The participants were selected purposively and the interviews were conducted at their time and place of convenience each not lasting more than one and a half hours. Sampling was guided by the principle of data saturation.

### Data Collection

Data were collected using In-depth interviews with supervisors and students. The guiding question was asking the participants to talk about what they knew about this method of learning called practice-based learning along with several probes. The probes included asking the participants to talk about their views about it and how they feel about their expectations. We also probed for challenges, skills gaining, self-efficacy, improvements, and suggestions. All interviews were conducted by the principal investigator with assistance from two experienced research assistants who took notes during the interview. The interviews were conducted in English and participant responses were captured using an audio recorder. The participants were allowed to withdraw from the study at any time.

### Data Management and Analysis

Thematic analysis was employed. The audio recordings from the interviews were transcribed and later imported into Atlas ti 8.3 for analysis. The data were coded and grouped into themes relating to perceptions of practice-based learning, general inductive analysis was used [15]. The general inductive approach involved condensing the raw textual data into a brief and summary format. The summarized format was then analyzed to establish clear links between the perceptions of practice-based learning and the summary findings derived from the raw data. Thereafter, a framework of the underlying structure of perceptions that are evident in the raw data was developed [15].

### Quality control

Qualitative validity was determined through the use of strategies to check the accuracy of the findings and these include credibility, transferability, dependability, and confirmability [16]. Credibility was achieved through prolonged engagement, peer debriefing, triangulation, and member checks [16]. Furthermore, participants were engaged till the principal investigator had richly collected data to answer the objectives. During peer debriefing, the principal investigator shared the protocol, data collection tools, and the write-up with other peers to get their perspectives in a bid to check for general errors in the data, vague descriptions, overemphasized points, and underemphasized points. Transferability was emphasized by providing thick descriptions of the themes and purposive sampling. Dependability was established with the audit trail which involved maintaining and preserving all transcripts, notes, audio files, and notes that were used to collect and analyze data. The principal investigator also ensured that participants member checked their transcripts while also probing for additional information about the phenomenon of interest. Conformability was determined by linking the data to their sources through triangulation from the data collected from interviews, observations made at the study sites, and review of journal articles, and reports among other documents from the web to create a comprehensive understanding of particular phenomena [17].

## Results

### Description of study participants

The mean age of the students was 23 ± 1.5 years. Four out of the six supervisors were Orthopaedic officers while the remaining two were principal Orthopaedic officers, four out of the six had a university degree while the other two were diploma holders.

### Orthopaedic students and supervisor perceptions about practice-based learning

In this section, we present the major themes arising from the analysis. Analysis of data resulted in 4 major themes namely: (1) hands-on skills, (2) an unconducive learning environment, (3) the best form of learning and (4) an undefined training structure. Most importantly to note is whereas the first two themes i.e., hands-on skills and unconducive learning environment were reported by the students, the best form of learning as well as the undefined training structure were reported by their supervisors. These themes are further explained along with supportive participant quotations.

### Hands-on skills

Students perceived practice-based learning as a very good method of learning that provides skills, a chance to practice what is taught, and gain confidence to manage patients. Some students explained;

“*It’s a very good method of learning because it gives us experience about how to handle patients, basically in our course, we need hands-on.”***(IDI/10)**.

another one explained;

“*I get that chance of doing what I am supposed to do as far as my course is concerned, I get that kind of exposure and practice.”***(IDI/07)**

while the other student explained;

“*If you practice something over and over again you get more experienced and confident.*” **(IDI/06)**.

### Unconducive learning environment

A large number of students reported to have encountered instances of inadequate training while onwards and being stressed during the training. This was enlightened by the interviews as some students perceived their supervisors to be harsh, they faced problems of overcrowding onward, inadequate placement time, and lack of materials and equipment to use. They explained;

“*…the area is so small, in that sometimes you are sent several people and the area are so small so all of you cannot see, may not see what is being done*.” **(IDI/01)**.

another one explained;

*“…. we face a challenge of inadequate supervision those who manage to come are busy*.” **(IDI/03)**

he continued to explain;

.. *some supervisors are rude, instead of teaching us they condemn you in case something is mistakenly done*.

About the inadequate materials and equipment, some students explained;

“*They should also provide enough equipment on the ward because we risk our lives a lot like acquiring infections due to absence of gloves.”***(IDI/04)**.

another one explained;

*“… most of the time the facilities are not enough for emergency patients like medicine which isn’t enough compared to the patients we receive, gloves aren’t enough, and* Orthopaedic *facilities are also not enough.”***(IDI/06)**

### The best form of learning

Interviews with the supervisors helped to reveal why most of them thought students are not gaining adequate skills and why most students express anxiety or stress during the training. The supervisors perceived practice-based learning as being advantageous, the best form of learning that provides experiences to students. They explained;

***“…****this it’s the best form of learning because these jobs are practical and they help a lot of students except for a few who aren’t interested because they do exist*.” **(IDI/T04)** he continued to explain;

…. *Practice-based learning is knowledge and skills you gain with hands-on, when a student is both working and learning at the same time, it involves picking up experience, the student work and we recess them*.

Another supervisor explained;

“*It carries the biggest part it is very advantageous much as theory is vital but theory alone cannot teach a student, so the practical skills they get especially here onwards is what carries the biggest part of their learning it is very vital to me that is it*.” **(IDI/T01)**

### Undefined training structure

Amongst the student supervisors, practice-based learning was faced with challenges of inadequate materials for the students, overcrowding, short placement time which affects the learning environment, and skills acquisition for the students. The supervisors explained;

*“… the students come in big numbers and end up not attended to but if they come in small numbers it won’t be an only advantage to the supervisor because he will attend to them with close supervision but also the students will have a chance of having practical work and utilizing the few materials and infrastructures available*.” **(IDI/T03)**

another one explained;

*“The hospital is being flooded by students from other private students from different parts of the world, they can be so many and the teaching becomes hard for us and we aren’t catered for.”***(IDI/T04)**

About the materials and equipment, one supervisor explained;

*“.. challenges the students face sometimes they are cases to manage but they are no materials to use so you find a patient has been kept on the ward for two days before he has been worked on so in that case if a student was going to participate in the management of that condition he misses out.”***(IDI/T02)**

Another supervisor explained’

*“Sometimes materials are limited we may not have certain working to use so we end up improvising much as we would have wanted them to know how to use what type of appliance*.” **(IDI/T03)**.

And about the short placement period, one supervisor explained;

*“…. to me these programs it’s like they are sent to the ward when teachers are busy doing other things then they say go to the ward but we don’t see them much as this is three years post they finish three years and you have never seen them yet am in a vital unit of accident and emergency so the time I think it’s not enough or them I can’t be exact but they stay for two weeks at most.”***(IDI/T01)**

## Discussion

This study aimed to explore the perceptions of Orthopaedic medicine students and their supervisors about practice-based learning in the emergency ward at Mulago National Referral Hospital. Four major themes namely: (1) hands-on skills, (2) unconducive learning environment, (3) best form of learning, and (4) undefined training structure were the perceptions that were identified in this study. In this study, both the students and the supervisors think of practice-based learning as an important and relevant method to gain insight into the Orthopaedic medicine discipline as portrayed by the themes of hands-on skills and the best form of learning theme. Still, there are issues and problems with the way the learning is organized, and this must be improved. This is demonstrated by the unconducive learning environment as well as the undefined learning structure.

Indeed, the challenges such as inadequate training space, a limited number of supervisors, unavailability of training materials, and a short training period would have a direct negative impact on the overall outcome of practice-based learning. The challenges identified (inadequate training space and a limited number of supervisors) could be dealt with by attaching Orthopaedic students to other facilities so that the ratio of the supervisor to trainee ratio is reduced. Similar results have been observed in students of family medicine in India, practice-based learning was reported to be 69% unsuccessful [18].

High levels of stress and anxiety have been reported to have a negative impact on students’ learning ability, performance and decision making [19]. In our study, some of the Orthopaedic students reported stress and anxiety during the training and others reported to have encountered insufficiency in managing patients. The cause of the anxiety to students can be explained by the harsh environment created by the supervisors as students reported in the interviews.

It has previously been reported that medical schools tend to preselect for individuals who are more perfectionists [20], and as such, this personality makes individuals vulnerable to anxiety [21]. Factors like academic workload [22], sleep deprivation [23], financial burden [23], exposure to deaths of patients [24] and student abuse [25] have as well been documented to be the reasons for anxiety. Issues related to student anxiety could be modified by developing favorable study schedules to allow the students sufficient time to do practice-based learning as opposed to academic based workload. Similar results have been reported among students of Birmingham [26] and Michigan [27].

Self-responsibility has been reported to impact on the skills gained during practice-based learning, students who take self-responsibility during management of patients acquire more skills and knowledge [28]. In our study, the participants objectively reported taking self-responsibility as pertinent. Although the students have self-responsibility in managing patients, other factors like overcrowding on wards by patients and students limit their hands-on skills gaining and acquiring knowledge. Similar results have been reported in the United Kingdom (Britain), 1993) and in South America [29].

Attitudes towards learning also affect a student’s learning ability, negative attitudes towards practice-based learning will constrain student’s acquisition of knowledge and skills [30]. In our study, participants had positive attitudes towards practice-based learning as they described it as the best way for hands-on practice and practical learning abilities. Despite the positive attitude towards the method of learning, students have encountered other factors that limit their learning abilities. Similar results have been described by Eraut, 2004.

Placement time was reported to be insufficient by students and supervisors and it impacts on the skills they gain and general competence. As a result, the students reported to have encountered times of insufficiency in managing patients and they suggested to have longer periods of placement for enough exposure. Inadequate placement time could have been so because it runs concurrently with the academic schedule making it difficult to prolong the training time. In relation to the harsh supervisors, the school departments should develop platforms through which students can give feedback on abusive and unreliable medical supervisors to minimize psychological torture imposed onto the trainees. Similar results have been reported among residents of India [18] and nursing students in Sweden [31]. Longer placement time provides more opportunities to the students to learn and get more skills as they manage different cases [31].

Hospitals that are well equipped have been reported to produce students who have more skills than those which are not well equipped. Educational environment of students affects their learning abilities [32]. In this study, most students and their supervisors reported inadequate materials and equipment at the hospital. Many students are forced to share with the little equipment that is available and some of the unavailable equipment limits their scale of learning. This inadequacy makes student to gain limited skills as they are expected.

Supportiveness of the working environment has also been described by Nagraj et al., 2006 as a factor for students to gain skills and necessary knowledge [32]. In our study, the students reported the environment to be supportive. However, some students reported that supportiveness is lacking and more in terms of supervision as the number of human resources is low compared to the students. The students also described the attitudes of supervisors and nurses as being negative towards them which impacts on their learning abilities. Similar results have been reported among students in Ireland [33] and among students of Toronto [34].

The study utilized a qualitative method approach which gave a voice to study participants and ensured that study findings are grounded in participants’ experiences. The study also included both the Orthopaedic students and their supervisors. This therefore contributed to the validity and strength of the study findings. There are key implications of these findings to practice-based learning. The findings suggest that if institutions deploy fewer students matching with the few supervisors on ward per rotation and for longer placement periods, students will gain more hands-on experience and skills.

There are also key implications for further research. For example, we would recommend more research to be conducted on how to improve students’ learning abilities, the barriers and facilitators to practice-based learning.

The study had some limitations. The qualitative responses from students and supervisors were based on subjectivity (self-reports) which could limit the truthfulness and hence information bias. Participants who were selected could have been those who were available at that particular time when they were supposed to be there since data was collected within a period of just one month. This may have been an agent of selection bias in this study. However, despite these limitations, this study still provides key valuable insights into practice-based learning upon which more studies in different settings can be built.

## Conclusion

The status of practice-based learning as perceived by respondents was unsatisfactory. Hands on skills, unconducive learning environment, best form of learning and undefined training structure were the major perceptions identified in this study. More efforts are required to improve practice-based learning if it is to be successful, hospitals need to be more equipped and provided with materials. The institutions should deploy fewer students per rotation and for longer placement periods to match with the few supervisors on ward to enable students gain more hands-on experience and skills. The supervisors should engage in more student supervision, provide timely feedback and have a positive attitude towards students. Further research should focus on how to improve students’ learning abilities, the barriers and facilitators to practice-based learning.

## Data Availability

All qualitative data generated or analyzed during this study are available from the corresponding author on reasonable request.

## Abbreviations

ICT: Information Communication and Technology
IDI: In-depth Interview
